# Off-Label Use of Botulinum Toxin in Dermatology—Current State of the Art

**DOI:** 10.3390/molecules27103143

**Published:** 2022-05-13

**Authors:** Miłosz Lewandowski, Zuzanna Świerczewska, Wioletta Barańska-Rybak

**Affiliations:** Department of Dermatology, Venereology and Allergology, Faculty of Medicine, Medical University of Gdansk, Smoluchowskiego 17, 80-214 Gdansk, Poland; milosz.lewandowski@gumed.edu.pl (M.L.); zuzanna.swierczewska@gumed.edu.pl (Z.Ś.)

**Keywords:** botulinum toxin, off-label use, dermatology, hidradenitis suppurativa, review, rosacea

## Abstract

Botulinum toxin (BoNT) is a neurotoxin produced by the *Clostridium botulinum* bacteria. Among seven different isoforms, only BoNT-A and BoNT-B are commercially used. Currently, botulinum toxin has been indicated by the U.S. Food and Drug Administration in several disorders, among others: chronic migraine, hyperhidrosis, urinary incontinence from detrusor overactivity, or cosmetics. However, there are numerous promising reports based on off-label BTX usage, indicating its potential effectiveness in other diseases, which remains unknown to many. Among them, dermatological conditions, such as rosacea, annal fissure, Raynaud phenomenon, hypertrophic scars and keloids, and also hidradenitis suppurativa, are currently being investigated. This article aims to provide a comprehensive update on the off-label use of botulinum toxin in dermatology, based on an analysis and summary of the published literature.

## 1. Introduction

Botulinum toxin (BoNT) is a neurotoxin produced by the *Clostridium botulinum* bacteria and occurs as the following seven different isoforms: BoNT-A, B, C, D, E, F, and G, among which BoNT-A and BoNT-B are the only commercially used. Botulinum toxin application in medicine as a therapeutic option was first mentioned in the literature by AB Scott as a case report study, showing significant improvement of BoNT in strabismus treatment. The U.S. Food and Drug Administration approved botulinum toxin for the first time for the treatment of strabismus in 1989 [1]. Since then, the BoNT has been indicated in several disorders, among others, chronic migraine, hyperhidrosis, urinary incontinence from detrusor overactivity, or cosmetics [2]. There are numerous promising reports based on off-label BoNT usage, indicating its potential effectiveness in other diseases, which remains unknown to many. Among them, dermatological conditions, such as rosacea, annal fissure, Raynaud phenomenon, and also hidradenitis suppurativa, are currently being investigated [3,4,5,6]. The aim of the review was to analyze and summarize published literature on the future possible indications for the use of botulinum toxin in dermatology.

## 2. Mechanism of Action of BoNT

BoNT-A and BoNT-B are di-chain polypeptides composed of a heavy chain and a light chain linked by a disulfide bond. For both A and B, the heavy chain is responsible for the selective binding of the neurotoxin to cholinergic nerve terminals, while the light chain inhibits acetylcholine release [7]. At this point, these types A and B differ. The light chain of type A cleaves SNAP-25 protein, while the light chain of type B cleaves vesicle-associated membrane protein (VAMP) [8]. The well-established mechanism of action of BoNT is to inhibit acetylcholine (Ach) and other neurotransmitters released at the presynaptic neuromuscular junction by deactivating SNARE proteins. Thanks to its action, BoNT may block both sympathetic and parasympathetic nerve terminals [9].

## 3. Safety of BoNT

Botulinum toxin therapy is usually regarded as safe, effective, and relatively free of major adverse effects. Pain, edema, erythema, ecchymosis, and short-term hypesthesia are the most common possible side effects of botulinum toxin injections [10]. Serious adverse events may also occur; however, they are significantly less frequent than mild ones. It has been reported that serious side effects of BoNT are more often observed for therapeutic rather than for cosmetic use, which is probably related to the median botulinum toxin dose, four times higher for therapeutic than for cosmetic indications [11]. Dysphagia, muscular weakness, and allergic responses are among the serious side effects of botulinum toxin cosmetic usage [11]. The other severe one may be botulism, which is dose-dependent and caused by botulinum toxin diffusion into nearby muscles from the injected muscles. In a clinical analysis, Lili Bai et al. observed botulism manifestations in 86 patients after cosmetic injections of botulinum toxin [12]. These were observed as headaches, dizziness, insomnia, fatigue, blurred vision, difficulty with opening eyes, slurred speech, dysphagia, bucking, constipation, or anxiety, depending on the botulism severity [12]. Its main therapy is based on the administration of neurotrophic medicines, symptomatic therapy, as well as the injection of botulinum antitoxin serum [13].

## 4. Role of BoNT in Dermatology

### 4.1. Hidradenitis Suppurativa

*Hidradenitis suppurativa* (HS), also known as *acne inversa*, is a chronic, progressive, debilitating, recurrent inflammatory skin disease characterized by the occurrence of very severe, persistent, painful nodules, abscesses, and fistulas, most commonly found in the skin folds of the axilla, groin, gluteal, and perianal areas [14]. Although the pathogenesis of hidradenitis suppurativa is still being investigated, it is currently considered to be an immune-mediated inflammatory illness (IMID) [15]. The etiology of the disease is multifactorial, with genetic, hormonal, immune-regulation, lifestyle, and dysbiosis, all likely contributing to pilosebaceous unit hyperkeratinization and occlusion, which leads to perifollicular lymphocytic-histiocytic inflammation [16]. Eventually, the hair follicle ruptures, followed by the release of follicular contents into the dermis [17]. Despite multiple options available, the treatment of the disease is often challenging.

In a randomized, double-blind, placebo-controlled pilot study by Grimstad et al., the effectiveness of BoNT-B in 20 patients with hidradenitis suppurativa stage I–III disease was investigated [5]. Among the study group, 55% of patients reported concomitant hyperhidrosis, as evaluated by the validated Hyperhidrosis Disease Severity Scale (HDSS). The study participants (*n* = 20), randomized in a 1:1 ratio, received either the BoNT-B or placebo. After 3 months, both of the groups received treatment with BoNT-B. Depending on the location of the lesions, patients were injected with variable dosages of the active substance. Since two study groups received either one or two BoNT-B treatments, the differences in each group separately with changes from baseline to the evaluation at 3- and 6-months post-treatment were calculated. After BTX-B injections at follow-up in 6 months, a significant improvement in the total number of skin lesions was observed in both groups. Depending on the location of the lesions, patients were injected with variable dosages of BoNT-B. Additionally, a significant improvement in the Dermatology Life Quality Index (DLQI) at follow-up at 3 months and 6 months and a tendency for improvement in the DLQI were noticed in the BoNT-B completely treated group and the placebo-BoNT-B treated group, respectively. The authors concluded that injections with BoNT-B improve the quality of life as well as self-assessed symptoms in patients with acne inversa. What is more, comorbidity between HS and hyperhidrosis was suggested.

Thus far, several case reports describing the use of BoNT injections have been published. The first report of the management of acne inversa with botulinum toxin type A was in 2005 [18]. O’Reilly et al. reported successful use of BoNT-A injections in one patient with HS. A total of 250 units of a toxin were injected into axillary skin lesions, and after a sum of four procedures, a complete response with a 10-month remission was noted. Comparable results were also observed in two other studies on patients with moderate HS [19,20].

Shi et al. presented a case of a 41-year-old woman with stage III hidradenitis suppurativa after uneventful treatment with all available methods. The patient received BoNT-A injections every 3 months, for a total of four times. The botulinum toxin was dissolved with normal saline (100 units in 2 mL of normal saline) and injected intradermally, five units in 0.1 mL for each injection, about 20 injections in one area. An excellent response on follow-up examination was noticed, with a significant reduction in inflammation and drainage of some sinus tracts. The patient also reported overall at least 50% pain relief. No other treatments were applied during the botulinum toxin therapy [21].

Although relatively rare, prepubertal hidradenitis suppurativa is also present in the population. Feito-Rodriguez et al. presented a case of a 6-year-old girl with erythematous papules and nodules involving the groin area. However, the patient had previously been treated with topical antibiotics and oral isotretinoin without satisfactory results. At 7 years, a total dose of 40 IU of BoNT was injected intradermally from 10 to 12 points over the affected areas. Complete remission of lesions until 6 months later was observed. The relapse responded as effectively to a second treatment as they had to the primary injection [22].

A summary of the use of BoNT-A in hidradenitis suppurativa is presented in Table 1.

### 4.2. Rosacea

Rosacea is a chronic, inflammatory skin disease marked by redness, erythema, telangiectasia, hyperplastic lesions, papules, and pustules. They are most often manifested in the area of the face around the forehead, nose, cheeks, and chin. The prevalence of the disease is estimated at 10% in the Caucasian population. Rosacea is significantly more common in women, and although its onset may occur at any age, most often the first symptoms appear after the age of 30 [23]. The pathogenesis of rosacea is multifactorial and still not fully understood, while the currently adopted model includes the influence of genetic, immunological, and environmental factors, as well as vasomotor disorders. The role of microorganisms such as *Staphylococcus epidermidis* or *Demodex folliculorum* is also important in the pathogenesis of rosacea [24]. The factors exacerbating the course of the disease include stress, UV radiation, alcohol, and some medications. Currently, there are many therapies available for the treatment of rosacea, but there is still a need for novel approaches that open up new avenues for the treatment of rosacea.

The mechanism of action of botulinum toxin is to inhibit the release of acetylcholine (ACh) from the presynaptic vesicle at the neuromuscular junction [25]. Moreover, it has the ability to modulate other neuropeptides, including substance P, the calcitonin gene-related peptide (CGRP), and the vasoactive intestinal peptide (VIP) [26]. The key mediators of vasodilation and redness are Ach and VIP; therefore, inhibiting their release seems to be a reasonable mechanism to explain the action of BoNT in rosacea. However, the exact mechanism by which botulinum toxin is effective in the treatment of rosacea remains unclear.

Choi et al. [6] attempted to investigate the molecular mechanism by which botulinum toxin shows the ability to improve symptoms of rosacea. Primary human and mouse mast cells were pretreated with botulinum toxins A or B. An in vivo model of rosacea was established by intradermal injection of LL-37 in mice with or without botulinum toxin A pretreatment. It was proven that botulinum toxin A and B inhibited the induced degranulation of both human and murine mast cells. In mice, injection of botulinum toxin A significantly reduced LL-37-induced skin erythema, mast cell degranulation, and mRNA expression of rosacea biomarkers. These results suggest that botulinum toxin may reduce skin inflammation by directly inhibiting mast cell degranulation, which, in turn, reduces the erythema associated with the course of rosacea.

Another promising study was conducted on 20 Korean patients with refractory rosacea of the erythematous-vascular type, in which the efficacy of intradermal botulinum toxin was assessed [27]. All patients in the study group received a total of 20 IU of botulinum toxin A, and the injection points were staggered 1 cm apart to cover the erythematous lesions of both cheeks with the use of a 30-gauge insulin syringe. The subjects were then assessed at follow-up visits at 1, 2, 4, and 8 weeks after the treatment. The parameters assessed were the severity of erythema and telangiectasia, as well as patient satisfaction and any adverse events. Of the 20 patients, 17 completed the study. It was shown that the intradermal injection of botulinum toxin significantly reduced the severity of erythema in patients included in the study. With the exception of three patients who discontinued the study due to midfacial paralysis, no adverse events, apart from the pain associated with the injection of a toxin during the procedure, were reported in the final analysis based on data from 17 patients.

In another double-blind, randomized trial conducted on 24 patients with rosacea, 15 IU of BoNT-A was injected on one cheek and saline on the other [28]. Two months after the procedure, a significant improvement in erythema, elasticity, and skin hydration was observed on the side treated with BoNT-A. Similar results were obtained in a study conducted on 9 patients with rosacea by Dayan et al. [29]. Persistent improvement in acne rosacea was observed in the botulinum toxin treatment group 4 weeks after treatment. After 16 weeks, both groups received BoNT-A treatment, showing a significant improvement in rosacea-related symptoms as well as an increase in patient satisfaction index.

Recently, Tong et al. [30] tried to assess the efficacy and safety of BoNT combined with broadband light (BBL) in the treatment of rosacea-related erythema and flushing. The authors enrolled 22 subjects with erythematotelangiectatic rosacea. Both cheeks were randomly divided into the study and control groups and received treatment three times. During the first treatment, the study group was treated with BBL and an intradermal injection of BoNT, and the control group received BBL treatment and an intradermal injection of the same amount of saline. During the second and third treatments, both groups received the same BBL therapy. In the study group, an improvement in skin hydration along with a reduction in flushing, erythema, transepidermal water loss, and sebum secretion was noted in comparison with the controls. A conclusion was made that BoNT injection combined with BBL has a high safety profile and efficacy in the treatment of rosacea-related erythema and flushing, better than BBL itself.

A summary of the use of BoNT-A in rosacea is presented in Table 2.

### 4.3. Anal Fissure

An anal fissure is one of the most common anorectal conditions worldwide. It is defined as a distally spreading linear tear in the mucosa of the anal canal, usually reaching from the dentate line to the anal margin [31]. According to the current literature, it is estimated that 90% of them are located in the posterior midline [32]. CAF’s pathophysiology has not been fully understood yet. It is believed that hypertonia and spasm of the internal anal sphincter (IAS), leading to local ischemia, have a significant influence on the development of chronic anal fissures [33]. There are several options for its management [34]. The Association of ColoProctologists of Great Britain and Ireland, the American College of Gastroenterology, and the American Society of Colon and Rectal Surgeons currently recommend diltiazem as first-line therapy for CAF. It is preferable over topical nitrates (Topical Glyceryl trinitrate, GTN) because of the fewer side effects caused by calcium blocker drugs [35]. According to the mentioned guidelines, BoNT may be used as a second-line therapy for fissure resistance to topical GTN or diltiazem. A systemic review of randomized controlled trials by P. A. Boland et al. underlines that despite almost equal healing rates at 8 weeks between the BoNT and nitrate groups, 66.7% and 63.8%, respectively, and a slightly lower 52.3% healing rate for topical diltiazem, BoNT treatment demonstrated the highest recurrence rate at 41.7% and high fecal incontinence at 14.4%, compared to 10.0% for sphincterotomy and 1.9% for topical nitrates [36].

Among the mentioned guidelines, only The Association of ColoProctologists of Great Britain and Ireland suggests a specific botulinum toxin dose of 20–25 IU in two divided doses injected into the internal sphincter on either side of the fissure [35]. However, recommendations from the Association of Coloproctology of Great Britain and Ireland, as well as a meta-analysis by Adam Bobkiewicz et al., underline that BoNT management efficacy is not dose-dependent, and the rate of postoperative fecal incontinence is unrelated to the BT dosage. Moreover, there is no difference in the rates of healing depending on the sites and the number of injections per session [4]. In the other meta-analysis, the authors analyzed statistical variables associated with BoNT anal fissure treatment over a longer period of time compared to the previously mentioned study, 3 months vs. 2 weeks, respectively, and also declared no correlation between dosage and treatment effects. Nonetheless, they concluded that lower BoNT doses slightly reduce the risk of incontinence and recurrence in the long term [4].

It is worth noticing that in the survey study at the turn of the year 2019/2020, conducted on members of the American Society of Colon and Rectal Surgeons (ASCRS), 90% of all 216 respondents declared injecting BoNT in 50–100 IU doses, which does not correlate with the statement of the Association of Coloproctology of Great Britain and Ireland (ACPGBI). Moreover, a majority of them (53%) inject into four quadrants in the anal canal circumference [37]. The discrepancy between guidelines and practice shows that further research is needed to develop appropriate anal fissure treatment standards with BoNT usage.

### 4.4. Raynaud Phenomenon

The Raynaud phenomenon (RP) is an exaggerated reaction of the extremities to cold or emotional stress that is amplified. The following two types can be distinguished: the primary Raynaud phenomenon, which is a benign disorder induced by alterations in the function of blood vessels and/or their innervation that do not develop into irreparable tissue harm, and the secondary Raynaud phenomenon, which is a result of comorbidities such as systemic sclerosis (SSc), systemic lupus erythematosus (SLE), vasculitis, atherosclerosis, and hypothyroidism [38]. It is underlined that despite the relatively high prevalence (4.85%) and annual incidence (0.25%) of the disease, there is still no international consensus published regarding its management [39,40].

The current guidelines of the European Society of Vascular Medicine from 2017 recommend starting the management of the Raynaud phenomenon with a patient’s lifestyle modifications, among others, protecting from the cold, smoking cessation, and avoiding vibration exposure. For those who did not respond to these general measures, drug treatment or surgery are reserved [40]. According to the guidelines, the first line of medicine is calcium channel blockers, e.g., nifedipine, but the second-line drugs include other vasodilators such as angiotensin receptor antagonists or intravenous prostaglandins. Surgery is not indicated in the primary RP; however, it may be necessary for secondary RP patients who have developed digital ulcers and/or gangrene. In secondary care for digital necrosis, surgical debridement/operations to remove portions of the terminal phalanx or amputation are required [40]. Conventional treatments are not always effective, making it necessary to look for alternative therapies, which may include the botulinum toxin. It is crucial to emphasize that despite the good efficacy presented in various papers, botulinum toxin is not even mentioned in the European Society of Vascular Medicine guidelines.

The exact mechanism of BoNT-As action is uncertain, although it probably causes more than just vasodilation by paralyzing acetylcholine-mediated arterial muscles [41]. The majority of single original studies, including prospective studies, demonstrated the good effectiveness of BoNT-A in relieving vasospasm and alleviating pain in patients with RP [42,43,44,45,46,47]. However, a randomized controlled trial published in 2017 by Ricardo J. Bello et al. showed that laboratory-based LDI flow data do not support using BoNT-A to treat RP in all scleroderma patients, presenting significant improvement in blood flow from baseline to 1-month follow-up in BoNT-A group compared to a placebo, however, this significant phenomenon was not observed at 4-month follow-up. Hands in the study group received 50 IU of BoNT-A in total using a 30-gauge needle, reconstituted in 2.5 mL of sterile saline. Hands in the control group received 2.5 mL of saline in total as a placebo. What is more, a comparison result between the control and placebo group in QuickDASH score (hand function), McCabe Cold Sensitivity Score, VAS pain scale score, and the number of active ulcers was not statistically significant at the 4-month follow-up [48]. In the systemic review performed, including all studies that contained reports of botulinum toxin A use and its outcomes in Raynaud’s phenomenon, Żebryk et al. underlined that there is still insufficient evidence to assess the efficacy of botulinum toxin A in Raynaud’s phenomenon [49].

Despite the not fully satisfactory results of both of the above-mentioned studies, each of them concludes their worth and needs to investigate further advanced research, among other randomized controlled trials, in order to investigate BoNT-As effectiveness for Raynaud’s phenomenon management.

### 4.5. Androgenetic Alopecia

Androgenetic alopecia (AGA) is the most prevalent kind of non-scarring alopecia, affecting up to 80% of men and 50% of women during their lives, with a frequency that rises with age following puberty [50]. Nowadays, the topic of androgenetic alopecia is becoming more and more important because hair has become a significant part of one’s image in society, and thick, healthy hair is often connected with youth, attractiveness, and success. The most currently recommended therapies for AGA include the following: Finasteride 1 mg once daily (in men, first level of evidence); Minoxidil 5% twice daily (solution, foam) (in men, first level of evidence); Minoxidil 2% twice daily solution or Minoxidil 5% once-daily foam (in women, first level of evidence); hair transplantation with/without combination treatment (second level of evidence for men and fourth for women); low-level laser therapy (second level of evidence for women and men). One of the interventions mentioned in the most current European Dermatology Forum guidelines from 2017 is botulinum toxin.

According to the recommendation, based on only one clinical trial, by Freund and Schwartz from the year 2009, despite the promising results of the study, there is insufficient evidence for therapy with Botulin toxin injection in patients with androgenetic alopecia (Level of evidence—4, Grade of evidence—C) [51]. Moreover, the potential use of botulinum toxin was not even mentioned in the guidelines of the Japanese Dermatological Association [52]. Since the release of the guidelines, two clinical trial studies have been published. The first one, by Yaguang Zhou et al., was conducted on 63 patients divided randomly into BoNT-A (*n* = 30) and BoNT-A+Finasteride(FNS) (*n* = 33) groups. The authors observed the effective rates, after four times of therapy, for BTA and BoNT-A+Finasteride, at 73.3% and 84.8%, respectively, concluding that BTA may be a safe and effective therapeutic strategy for the treatment of AGA without adverse effects and that BTA combined with FNS exhibited a superior therapeutic effect than BTA alone. However, it is worth considering that there was no significant difference in the efficacy between BoNT-A and BoNT-A+FNS in patients with AGA [53]. In the mentioned study, a total injection dose of 100 U/mL was administered intramuscularly to the study group, and each injection site was 1.5-2 cm apart. The second research comprised 18 male patients treated intradermally with BoNT injections every 4 weeks for 24 weeks [54]. A total dose of 30 IU of BoNT was injected at 20 different sites on the balding scalp in each treatment session. At week 24, the number of hairs significantly increased compared to the pre-treatment condition (*p*= 0.012). A comparison of the pre- and post-treatment photographs showed significant improvement at week 24 (*p* = 0.031). Additionally, the authors conducted an in vitro study, suggesting inhibiting TGF-B1 secretion from the hair follicles by the intradermal injection of botulinum toxin may be the potential action mechanism of BoNT-A in treating AGA. Despite the promising results of the mentioned research, recently published systemic reviews underline that there is still a need to conduct randomized, placebo-controlled studies in this area [55,56,57].

### 4.6. Plaque Psoriasis

Psoriasis is a chronic, papulosquamous skin disease characterized by erythematous, scaly patches, or plaques. The most common variant, representing approximately 80-90% of all cases, is plaque psoriasis [58,59]. Typically, affected areas include the scalp, trunk, elbows, knees, and gluteal fold. Lesions vary from small, erythematous, and scaly papules to large, thick plaques. Treatment is usually based on topical agents such as corticosteroids or keratolytics, as well as phototherapy, oral systemic drugs, and biologics [59].

In research by Khattab et al., the efficacy and safety of BoNT and intralesional 5-fluorouracil were studied [60]. Thirty-five patients with chronic plaque psoriasis were either treated with intradermal BoNT or with intralesional 5-fluorouracil for each of two bilaterally symmetrical plaque lesions. The authors reported a response rate of 85% on the BoNT treatment side and 90% on the 5-fluorouracil side. No significant difference concerning clinical response or side effects was noted. On both sides, up to 15% of patients experienced a recurrence. Another study from 2020 has also shown clinical improvement of psoriasis plaques following BoNT injections in eight recruited patients [61]. What is more, two of the patients reported a significant decrease in the pruritus associated with their lesions. The authors reported no adverse effects related to BoNT injections, and none of the patients discontinued their participation during the study. A single case report by Gilbert et al. [62] also proved the efficacy of injections with botulinum toxin A. The described patient with refractory psoriasis received 30 IU of BoNT-A to a single lesional plaque and showed a complete remission for 7 months. The lesion recurred in the same location 8 months after the injection.

On the other hand, an exploratory, multicenter, randomized, double-blinded trial by Todberg et al. showed no effect of a single-dose BoNT-A injection [63]. In this study, 20 patients were enrolled; however, only eight of the planned 20 subjects were analyzed. In each case, two plaques were selected and randomized either for botulinum toxin therapy or sodium chloride therapy as a control group. BoNT was injected 0.04 mL in total. The authors concluded that a single dose of BoNT-A was not effective in clearing psoriatic lesions; however, more doses of BoNT could be more beneficial.

### 4.7. Inverse Psoriasis

Several reports have brought up the topic of BoNT in the treatment of inverse psoriasis. By reducing local sweat production and thus skin maceration, hyperkeratosis, inflammation, and pain, botulinum toxin may be efficient in the therapy of the disease. Zanchi et al. tried to evaluate the efficacy of botulinum toxin type-A for the treatment of inverse psoriasis [64]. Fifteen patients enrolled in the study received 2.4 IU BoNT-A, with a total dosage of between 50–100 IU per patient. All subjects reported decreased pruritus and pain after 3 months post-injection. Furthermore, intensity and infiltration improved in 87% of patients. The authors noted no serious side effects.

Another successful treatment with BoNT was noted by Saber et al. in a 26-year-old male patient with concominant axillary hyperhidrosis [65]. The patient reported improvement following bilateral injections with 100 IU of BoNT-A in the axilla. The first improvement in the psoriasis lesions was seen after one week post injection.

### 4.8. Hailey–Hailey Disease

Hailey–Hailey disease (HHD) is a rare autosomal dominant genodermatosis caused by a mutation of the *ATP2C1* gene, clinically manifesting as recurrent vesicular and erosive lesions involving intertriginous areas [66]. Factors that may exacerbate the disease include pregnancy, skin infections, menstruation, sweat, and heat [67,68,69,70]. Despite developments in the knowledge of the disease, management remains challenging, especially due to its chronic and refractory nature.

Several studies tried to assess the efficacy of BoNT in the treatment of HHD. The first to report a successful botulinum toxin A application in a patient with HHD was Lapiere et al. in the year 2000 [71]. The efficacy of BoNT-A injections was associated with a decrease in sweat production, thus reducing the risk of secondary infection and exacerbation. Subsequently, in an open-label pilot study by Dreyfus et al., 26 patients with Hailey—Hailey disease were evaluated for the effectiveness of BoNT-A injections [72]. The BoNT-A was diluted with 8 mL of sterile saline and, using a 30-gauge needle, 2 mL (50 units) was then administered, resulting in approximately 20 intradermal injections over the entire treated area. The target concentration was 50 IU per 100 cm^2^. Improvement was observed in 2/3 of patients after one month and remained during the 6-month follow-up period. Up to 37% of patients with the most severe HHD experienced a relapse during the study. This may suggest that in such cases, BoNT-A has to be used at higher doses or in combination with other therapies. Additionally, no serious side effects were observed.

Another study by Charlton et al. reported a severe HHD case with painful, blistering plaques and fissures in his axillae and groin that was previously treated without satisfactory effects [73]. The patient was administered 50 IU of BoNT—A at each site in his axillae and groin area. A reduction of symptoms to two episodes a year was observed. Furthermore, one study compared the effect of BoNT to that of laser ablation and dermabrasion [74]. The authors proved that botulinum toxin alone turned out to be efficient in inducing remission of HHD for at least 12 months. In 2019, Kothapalli et al. [75] suggested injections with BoNT as a first-line treatment for Hailey–Hailey disease.

### 4.9. Eccrine Nevus

Eccrine nevus is a rare, benign skin hamartoma that typically occurs in childhood and adolescence [76]. Typically, it appears on the forearms without any skin anomalies; however, with localized hyperhidrosis. The treatment depends mostly on the size of the lesion, concomitant symptoms, and personal preferences. Surgical excision is recommended for small lesions, while greater nevi with accompanied hyperhidrosis can be treated with anticholinergic medications or BoNT.

Martin-Gorgojo et al. reported a case of a 16-year-old female patient with excessive sweating on the left forearm and the dorsal aspect of the left hand [77]. Multiple intradermal injections of BoNT-A into the affected area were applied with satisfactory results. Anhidrosis was maintained for 7 months after a follow-up. Another case report of a 35-year-old man also confirmed the effectiveness of botulinum toxin in the treatment of eccrine nevus [78]. The patient was injected with 100 IU of BoNT-A in total and a full response was noted 3 weeks post-injection that lasted for the following 9 months. What is more, Honeyman et al. presented a 12-year-old girl with congenital eccrine nevus successfully treated with BoNT-A [79].

### 4.10. Oily Skin

Oily skin is one of the most frequent complaints during a dermatological consultation. Such patients may also report enlarged pores or acne. Recently, the efficacy of injections of BoNT in the management of enlarged facial pores and seborrhea has been a topic of interest for many scientists. Aside from a decrease in sebum production, patients treated with BoNT injections commonly report an enhancement in overall skin texture, mostly due to a reduction in pore visibility. A study by Wu et al. [80] showed that intradermal injections of BoNT-A result not only in a decrease in sebum production but also in a decrease in the number of noticeable facial pores. Liew confirmed the findings by demonstrating a decrease in pore size 6 weeks after intradermal injections of BoNT-A [81]. A split-face controlled study performed on 20 patients by Sayed et al. [82] proved that intradermal injections of BoNT-A have the ability to decrease pore size and sebum production with satisfactory results lasting for an average of four months. In another study by Rose et al. [83], 25 patients with facial seborrhea were treated with 30–45 IU BoNT-A injections in the forehead area. Significantly lower sebum production at the injection site was noticed, with 21 patients reporting a high degree of satisfaction. According to a randomized, double-blinded, placebo-controlled study by Kesty and Goldberg [84], patients who received either 30 or 45 IU of BoNT-A showed statistically significant decreases in sebum production compared to both the control group and the patients treated with 15 IU of BoNT-A. The results of the treatment were maintained for six months.

The exact mechanism by which intradermal botulinum toxin injections decrease sebum production is yet to be established. Hence, more research is warranted to determine the optimum dosages and injection techniques, as well as to establish the best candidates for such procedures.

### 4.11. Pompholyx

Pompholyx, also called dyshidrotic eczema, is a recurrent inflammatory vesicobullous skin disease affecting the palms or soles. In a prospective pilot study by Wollina and Karamfilov [85], patients were given topical corticosteroids on both hands in combination with intracutaneous injections of 100 IU of BoNT-A on the more affected hand. Six patients who completed the study reported decreased pruritus and vesiculation. What is worth mentioning is that the results were achieved quicker when using the combination therapy. According to several other studies, treatment with 100–162 IU BoNT-A per palm was found beneficial in reducing perspiration, pruritus, and vesiculation [86,87].

### 4.12. Darier’s Disease

Several studies have described the efficacy of botulinum toxin for the treatment of Darier’s disease (DD). Its first use in DD was reported in 2007 by Kontochristopoulos et al. [88]. A 59-year-old woman was injected with 50 IU of BoNT-A per submammary region, achieving sweat reduction and thus a decrease in pain, burning, itching, tightness, and malodor for 4 months after treatment. In another case report, a 22-year-old woman with Darier’s disease and concomitant intertrigo was treated with 40 IU of BoNT-A into each inguinal fold and 20 U per anal fold with satisfactory results [89].

### 4.13. Hidrocystomas

Hidrocystoma is a rare, benign, cystic lesion of the sweat gland which usually arises from apocrine glands. Botulin toxin injections proved efficacious in the treatment of hidrocystomas. A 56-year-old woman with multiple eccrine hidrocystomas received perilesional injections of 1 IU of BoNT-A every 40 mm into the affected area (a total dose of 60 IU) [90]. The procedure was well-tolerated, and after 14 days, the lesions had almost completely resolved. At the 4-month follow-up visit, no recurrence was noted. Gheisari et al. enrolled 20 patients in a prospective study to assess the efficacy and safety of intralesional injections of BoNT-A for the treatment of multiple eccrine hidrocystomas [91]. Each lesion was injected intradermally at the base with approximately 1.5 IU of BoNT-A. Over 75% of eccrine hidrocystoma lesions resolved without any scarring, with results lasting for the following 2–5 months. Two patients reported mild smile asymmetry, and one patient noted lagophthalmos roughly 5–7 days after injection that would resolve in 3 weeks.

In 2016, Bordeol et al. presented a case report of a 29-year-old male with multiple apocrine hidrocystomas successfully treated with BoNT-A [92]. The patient received perilesional and intralesional injections of 2 IU of BoNT-A 5 mm apart (a total dose of 20 IU) to the affected region. Six weeks post-injection, full flattening of the lesions and clinical improvement were noticed. The patient reported no adverse effects from the BoNT-A injections. At the 8-month follow-up, the patient received a second treatment of 20 IU of BoNT-A for the glabellar region due to a few remaining lesions. At the two-year follow-up, no new lesions were observed.

### 4.14. Notalgia Paresthetica

Notalgia paresthetica (NP) is a sensory mononeuropathy of the back, in particular the T2–T6 dermatomes, characterized by localized pruritus and pain with an associated demarcated, hyperpigmented macule or patch [93]. BoNT-A injections have been found helpful in the management of the disease, especially due to their antipruritic properties.

A randomized controlled trial by Maari et al. [94] assessed the efficacy and safety of BoNT-A in patients diagnosed with NP. Ten subjects received a maximum dose of 200 IU of BoNT-A intradermal injection; however, one patient withdrew from the study. The study showed no statistically significant difference in pruritus between patients treated with BTX-A and the control group 8 weeks post-treatment. Adverse reactions were mostly mild. Similar to the above-mentioned trial, a study by Perez-Perez et al. [95] was also not able to prove a long-lasting beneficial effect of BoNT-A injections.

On the contrary, Weinfield reported two cases with NP successfully treated with intradermal injections of BoNT-A [96]. The first patient was treated with a total of 16 IU of BoNT-A and remained asymptomatic for over 18 months post-treatment. The second patient was initially injected with 24 IU of BoNT-A and, after 18 months, she received another treatment of 48 IU due to persisting pruritus. Within a week, the patient reported no symptoms. Similar results were obtained by Datta et al., who reported a 58-year-old patient with NP [97].

### 4.15. Linear Immunoglobulin A Bullous Dermatosis (LABD)

Linear immunoglobulin A bullous dermatosis (LABD) is a rare autoimmune blistering disease affecting both children and adults. A single case report described the efficacy of BoNT-A for the treatment of LABD [98]. A 17-year-old patient received 50 IU BoNT-A to her left axilla; nonetheless, a decrease in disease severity and high patient satisfaction prompted treatment of the other axilla using the same modalities. The effects of the treatment remained for 6 months; however, due to satisfactory results, the patient requested to repeat the injections.

### 4.16. Hypertrophic Scars and Keloids

Keloid scars are a difficult clinical entity that is thought to represent the extreme spectrum of scar hypertrophy caused by a variety of pathophysiological processes, including fibroblast hyperactivity. The difference between hypertrophic scars and keloids is that keloids are characterized by no spontaneous regression over time and cause excessive tissue proliferation beyond the margins of the primary wound [99]. The current management of these medical conditions is mainly based on intralesional corticosteroid injections and cryotherapy, pressure therapy, and laser therapy [100]. Botulinum toxin A, by regulating the balance between fibroblast proliferation and cell apoptosis and by immobilizing the muscles, thus reducing the tension of the skin tissue during the healing process, seems to be effective in the aesthetic improvement of postoperative scars [101].

Over the last years, several studies, including these on humans, have shown that BoNT-A may have a good effect in preventing and treating scars [102]. According to a systemic review provided by Catrin Sohrabi et al. referring to keloid scar treatment with botulinum toxin, the BoNT-A with high evidence may be equal to triamcinolone in providing a short-term decrease in keloidal volume, height, and vascularity. Several level 1 and 2 studies have also suggested that botulinum toxin may be particularly effective in reducing the discomfort and itching associated with keloid formation [102]. According to the authors, there are currently just a few trials evaluating the efficacy of botulinum toxin in the treatment of keloid scars after surgery; thus, there is a need to conduct such research [102]. Promising evidence refers to treating hypertrophic scars with BoNT-A. Three clinical studies, including one double-blind randomized controlled trial, proved significant clinical and cosmetic improvement among patients treated with the BoNT-A [103,104,105]. In all mentioned studies, botulinum toxin was administered at 2.5 U/cm^3^ intralesional once a month for a total of 12 weeks, resulting in, among others, itching sensation score, erythema score, and patients’ satisfaction improvement [103,104,105]. It shows that despite the lack of strict indication of the use of BoNT-A in the treatment of hypertrophic scars and keloids, the method is promising and may play a significant role in treating them in the future.

### 4.17. Primary Hiperhydrosis

Hyperhidrosis (HH) is defined as excessive sweat production. Due to the diversity of research reports determining the prevalence of HH, it is currently difficult to estimate it. One of the most recent studies from 2016 considering the US population suggested approximately 4.8% of the general population, while the studies based on Canadian, Polish, German, Chinese, or Japanese populations suggest it as 12.3–16.7% [106,107,108,109]. Primary hyperhidrosis (PHH) is defined as idiopathic symmetrical bilateral excessive sweating that is not caused by other medical conditions or medication side effects. It most commonly affects the axillae, palms, soles, and craniofacial region, decreasing the patient’s quality of life. To qualify HH as a primary type, the first possible causes of secondary HH should be excluded. To diagnose PHH, excessive sweating has to be observed for more than six months and include at least two of the following variables: occurring more than once per week; present in patients younger than 25 years of age; a family history exists; sweating is bilateral and symmetric; sweating ceases while asleep; sweating severely affects the patient’s daily activities [110]. Aluminum chloride application or iontophoresis treatment are two typical first choices, both of which have a few side effects. When topical treatment strategies fail, the second-line, widely used, and the recommended therapy is BoNT injections in monotherapy or in combination with aluminum chloride [111].

The strict U.S. Food and Drug Administration indication for therapy with BoNT is “For the treatment of severe primary axillary hyperhidrosis that is inadequately managed with topical agents.” The agency underlines that the safety and effectiveness of BoNT for hyperhidrosis in other body areas and use in patients under 18 years of age have not been established. The only FDA-approved BoNT formulation for the treatment is BoNT-A. A dosage of 50 units intradermally, from 0.1 to 0.2 mL aliquots to each axilla evenly distributed in multiple sites (10–15) approximately 1–2 cm apart is advised using the dilution of 100 Units/4 mL with 0.9% preservative-free sterile saline [112]. Despite the indications, BoNT-A has been demonstrated to be effective for the off-label usage of other hyperhidrosis areas such as palms [113,114], trunk [115], soles [116], and craniofacial regions [117], provided that in treating palmar and plantar primary hyperhidrosis, higher BoNT doses are needed [116]. The treatment of PRR with BoNT-A usage is considered to be safe, and the main adverse events that may occur are injection site pain and hemorrhage, non-axillary sweating, pharyngitis, and flu-like syndrome [112].

A summary of proposed BoNT-A dosing schemes in selected diseases according to the guidelines or original research (clinical trials, prospective studies) is presented in Table 3.

## 5. Conclusions

The indications for botulinum toxin have evolved tremendously since its introduction in 1989. This review highlights the potential of the use of BoNT based on the latest studies that indicate botulinum toxin injections may be beneficial as an alternative method of therapy in treating, among others, hyperhidrosis, hidradenitis suppurativa, Raynaud phenomenon, or anal fissure. Currently, there are numerous studies suggesting the effectiveness of botulinum toxin in the discussed diseases; however, it should be emphasized that the majority of evidence is based on low scientific quality publications and more clinical trials need to be conducted. Despite many favorable utilizations, the use of botulinum toxin is not without ramifications. To serve patients with relevant therapy and reduce related complications, dermatologists should be aware of both on- and off-label applications of botulinum toxin. Undoubtedly, a consensus on the treatment protocol for each indicator should be a topic of interest for practitioners to standardize all regimens with specific doses of BoNT.

## Figures and Tables

**Table 1:** BoNT-A in hidradenitis suppurativa.

Author Name, Year and Reference	Type of Study	Number of Patients	BoNT-A Doses	Follow-Up	Retreatment	Results
O’Reilly DJ, 2005 [18]	Case report	1	250 IU total dose	10 months	None	Complete remission
Feito-Rodriguez M, 2009 [22]	Case report	1	40 IU total dose	6 months	Yes	Complete remission
Khoo ABS, 2014 [19]	Case report	3 with 1 described	200 IU total dose	3 years	Yes	Complete remission
Campanati A, 2019 [20]	Case report	2	50 IU/axilla 100 IU/groin	1 year	Yes	Improvement
Shi W, 2019 [21]	Case report	1	100 IU/affected area	N/A	Yes	Reduction in inflammation and drainage of some sinus tracts
Grimstad Ø, 2020 [5]	Randomized, double-blind, placebo-controlled pilot study	20	150 IU/axilla 200 IU/groin 600 IU/perianal area	6 months	Yes	Significant improvement of Dermatology Life Quality Index (DLQI)

**Table 2:** BoNT-A in rosacea.

Author Name, Year and Reference	Type of Study	Number of Patients	BoNT-A Doses	Follow-Up	Retreatment	Results
Dayan, 2017 [29]	Pilot, double-blind, placebo-controlled study	9	20 IU/cheek	4 weeks post-treatment	Yes	A significant improvement in rosacea-related symptoms, increase in patient satisfaction index
Park KY, 2018 [27]	Pilot study	20 with 17 completing the study	20 IU total dose	8 weeks post-treatment	None	A significant reduction of the severity of erythema
Kim MJ, 2019 [28]	A randomized, double-blind, placebo-controlled, split-face pilot study	24 with 23 completing the study	15 IU/cheek	12 weeks post-treatment	None	A significant improvement in erythema, elasticity, and skin hydration
Tong Y, 2022 [30]	A randomized, controlled, split-face study	22	6~15 IU/cheek	6 months post-treatment	Yes	An improvement in skin hydration, reduction of flushing, erythema, transepidermal water loss, and sebum secretion

**Table 3:** Proposed BoNT-A dosing schemes in selected diseases according to the guidelines or original research (clinical trials, prospective studies), which demonstrated the efficacy of BoNT-A.

Type of Study	Disease	Author Name, Year and Reference	Number of Patients	BoNT-A Doses
Guidelines of The Association of ColoProctologists of Great Britain and Ireland	Annal Fissure	Arnold Wald, 2014 [35]	-	20–25 IU in two divided doses injected into the internal sphincter on either side of the fissure
Survey study	Annal Fissure	Daniel J. Borsuk, 2021 [37]	194 of 216 (members of the American Society of Colon and Rectal Surgeons)	50–100 IU, a majority respondents injected into the internal sphincter and into 4 quadrants in the anal canal circumference
Clinical Trial	Androgenic Alopecia	Yaguang Zhou, 2020 [53]	30 (BoNT-A as a control group)	Total injection dose of 100 IU/mL intramuscularly, and each injection site was 1.5–2 cm apart. BoNT-A was diluted with 3 mL of 0.9% normal saline, injected every 3 months, 4 times in total
Clinical Trial	Androgenic Alopecia	Uri Shon, 2020 [54]	18	A total dose of 30 IU of BoNT-A was injected intradermal at 20 different sites on the balding scalp in each treatment session (every 4 weeks, for 24 weeks)
Prospective study	Inverse psoriasis	M Zanchi, 2007 [66]	15	Individual injections of 2.4 IU BoNT-A, 2.8 cm apart of each other, with a total dosage between 50 and 100 IU per patient depending on the extent and severity of the psoriasis
Open—label pilot study	Hailey–Hailey Disease	Dreyfus, 2021 [72]	26	The BoNT-A was diluted with 8 mL of sterile saline, 2 mL (50 units) was then administered, resulting in approximately 20 intradermal injections over the entire treated area
Randomized Controlled Trial	Oily skin	Khadiga S Sayed, 2021 [82]	20	The 100 IU was reconstituted with 5 mL saline solution (0.9% NaCl) to achieve a concentration of 2 IU/0.1 mL. 0.1 mL of the reconstituted BoNT-A was intradermally facial injected at 1-cm spacing. The total dose injected per patient was 10 IU
Prospective study	Oily skin	Amy E. Rose, 2013 [83]	25	300 IU of BoNT-A was diluted using 3 mL of bacteriostatic saline. The forehead area was injected intradermally with 30–45 IU of botulinum toxin. Ten injection sites were chosen, and 3–5 IU of botulinum toxin were injected at each point
Randomized Controlled Trial	Oily skin	Katarina Kesty, 2021 [84]	50	30 or 45 IU of BoNT-A injected into forehead
Clinical Trial	Pompholyx	U Wollina, 2002 [85]	6	100 IU of BoNT-A diluted with 2 mL physiological sodium chloride solution was injected intracutaneously in aliquots of 0.1 mL along the fingers and in the palms in combination with usage topical corticosteroids
Clinical Trial	Pompholyx	Carl Swartling, 2002 [86]	10	100 IU of BoNT-A was diluted with 1.0 mL of unpreserved saline, gives 10 units per 0.1 mL. 20 microliters of 100 IU/mL (2 units) were injected intradermally every 15 mm on the volar aspects of the palms and fingers. The total injected dose was a mean of 162 IU
Prospective study	Hidrocystomas	Mehdi Gheisari, 2018 [91]	20	A 300 IU BoNT-A was diluted with 4 mL of saline solution without preservative to achieve a concentration of 7.5 IU/0.1 mL. Up to 1.5 unit of botulinum toxin was injected intradermally at the base of each lesion to raise a visible wheal.
Randomized Controlled Trial	Notalgia Paresthetica	Catherine Maari, 2014 [94]	10	Injections of 0.1 mL (50 IU/mL) for every 1–2 cm^2^ of hyperpigmented area. If there was no hyperpigmentation, the pruritic area as delimited by the patient was injected (maximum intradermal dose of 200 IU BoNT-A)
Observational Study	Notalgia Paresthetica	L. Pérez-Pérez, 2014 [95]	10	Every vial of BoNT-A was reconstituted with 2.5 mL of normal saline (0.9%) and an insulin syringe was then used to inject 4 units (0.1 mL) at each injection point. The total injected dose was 48–56 IU in periscapular area
U.S. Food and Drug Administration Indications	Primary Hyperhidrosis Axilla	U.S. Food and Drug Administration, 2017 [112]	-	A dosage of 50 units intradermally, in 0.1–0.2 mL aliquots to each axilla evenly distributed in multiple sites (10–15) approximately 1–2 cm apart is advised using the dilution of 100 Units/4 mL with 0.9% preservative-free sterile saline
A double-blind, randomized Clinical Trial	Primary palmar hyperhidrosis	M Simonetta Moreau, 2003 [113]	8	BoNT-A was diluted in 0.9% saline solution to achieve a concentration of 2.5 IU per 0.1 mL. BoNT-A was injected intradermally in 28 ± 1 sites (mean ± SD) in each palm, in the same session
Prospective study	Primary palmar hyperhidrosis	Ana María Pérez-Bernal, 2005 [114]	69	100 IU of BoNT-A diluted with 4.0 mL of 0.9% sterile preservative free, normal saline added for a resulting dose per 0.1 mL of 2.5 IU), in intradermal injections at doses of 2.5 IU per site were spaced approximately 1.5 cm apart. A total of 80–100 U BoNT-A per palm was used and both palms were treated in the same procedure
Comparative Study	Primary hyperhidrosis in the trunk	Won Oak Kim, 2009 [115]	17	100 IU was diluted with 5 mL of 1% lidocaine, resulting in a concentration of 2 U of BoNT-A per 0.1 mL solution. Patients were given a total dose of 100–500 IU in the trunk area. Intradermal injections raised tiny wheals spaced approximately 1.5 cm apart.
A double-blind randomized controlled trial	Hypertrophic scars	Ahmad R. Elshahed, 2020 [103]	30	100 IU was diluted in 2 mL of sterile, preservative-free 0.9% saline to constitute a solution at a concentration of 5 U/0.1 mL. BoNT-A was administered once a month for a total period of 3 months. It was injected into the body of the scar. The dose was adjusted to 2.5 IU/cm^2^ of the lesion.
Prospective clinical study	Hypertrophic scars	Alhasan M Elhefnawy, 2016 [104]	20	An intralesional injection of BoNT-A diluted in 2 mL of sterile, preservative-free 0.9% saline to constitute a solution at a concentration of 4 IU/0.1 mL) was administered once a month for a total period of three months. The dose was adjusted to 2.5 IU/cm^3^ of the lesion, not exceeding 100 units per session.
Randomized Controlled Trial	Hypertrophic scars	Zhibo Xiao, 2009 [105]	19	BoNT-A was administered 2.5 IU per cubic centimeter of lesion once monthly for a total of 3 months.

## Data Availability

Not applicable.
